# Optimization of ZnAl/Chitosan Supra-Nano Hybrid Preparation as Efficient Antibacterial Material

**DOI:** 10.3390/ijms20225705

**Published:** 2019-11-14

**Authors:** Bi Foua Claude Alain Gohi, Hong-Yan Zeng, Sheng Xu, Kai-Min Zou, Binyao Liu, Xiu Li Huang, Xiao-Ju Cao

**Affiliations:** 1 Biotechnology Institute, College of Chemical Engineering, Xiangtan University, Xiangtan 411105, Hunan, China; claudefouabi@hotmail.fr (B.F.C.A.G.); xusheng2016@xtu.edu.cn (S.X.); kaiminzou1146349876@aliyun.com (K.-M.Z.); 15773228967@163.com (X.-J.C.); 2 Biology and Chemical Engineering School, Panzhihua University, Panzhihua 617000, Sichuan, China; hxl121600@163.com; 3 School of Materials Science and Engineering, Southwest University of Science and Technology, Mianyang 621010, China; lby5462@126.com

**Keywords:** ZnAl/CS hybrid, bi-titration method, antibacterial activity, optimization, CCD design, kinetic

## Abstract

The menace of antimicrobial resistance continues to increase and hence the need to discover new antibiotics, especially alternative and effective sources such as hybrid organic-inorganic, organic-organic materials, and other combinations. In this study, an antimicrobial hybrid supra-nano material was prepared by the bi-titration synthesis method of chitosan (CS) and ZnAl layered double hydroxide. Fourier-transform infrared spectrometer (FTIR), thermogravimetric and differential thermal gravimetric (TGA/DTG), ultraviolet-visible (UV-Vis), X-ray diffraction (XRD), and scanning electron microscopy (SEM) analyses indicated that the ZnAl/CS hybrid exhibited low crystallinity with high thermal stability. The results of ZnAl/CS characterization showed the characteristic properties of the individual components ZnAl and CS, indicating a successful preparation of the ZnAl/CS hybrid. The antibacterial tests revealed that the ZnAl/CS hybrid possessed an enhanced antimicrobial effect against both *Escherichia coli* (*E. coli*, MTCC 739) and *Penicilliumcyclopium* (*P. cyclopium*, AS 3.4513). Under the central composite design (CCD) of the response surface methodology (RSM) tool, the parameters of the hybrid synthesis reaction were optimized and the result obtained was as follows: reaction pH was 11.3, reagent Zn/Al ratio was 3.27, and chitosan concentration was 1.07 g/L. After optimization, it was found that the antibacterial activity of ZnAl/CS was strengthened against *E. coli* as evidenced by a widening of the inhibition zone of about 41.6%. The antibacterial activity of ZnAl/CS was mainly due to the reactivation of the antibacterial activity of CS associated with the release of Zn^2+^ and Al^3+^ metal ions in addition to ZnO, Al_2_O_3_, and ZnAl_2_O_4_ compounds resulting from the method of preparation.

## 1. Introduction

Microbial pathogens are one of the leading causes of human morbidity in the world [1]. Unlike pathogens, antimicrobial agents have saved many lives and contributed to the development of modern medicine over the past five decades [2]. In fact, it has been shown that antibiotics are very effective against most fungi and pathogenic bacteria. However, the growing resistance of microbes to a large number of antibiotics currently used has become a global health problem. Therefore, this implies an urgent need for new improved antimicrobial drugs with molecular technology, which are also strengthened to eradicate resistant strains.

Recently, the use of hybrid materials at the supra-nanometric scale is an increasingly used approach in the fight against antimicrobial resistance [3,4,5]. The construction of hybrid organic-inorganic materials is a rapidly growing field of materials chemistry, designed to produce advanced materials with improved structure and functionality [6,7,8]. Among the candidate materials for hybrid formation, researchers paid particular attention to layered double hydroxide LDH (ZnAl, inorganic) and chitosan (CS, organic). They are of particular interest not only due to their individual properties, such as null toxicity and allergenicity, good thermal stability, biocompatibility, intercalation, and ion exchange, but also for their ability to exhibit an excellent combinationof physical, chemical, and mechanical properties in addition to being cost-effective [9,10,11]. ZnAl and CS have lately been the focus of attention in a variety of areas including chemical engineering, materials science, physics, and biomedical sciences [12,13,14,15]. In particular, CS, an abundant biopolymer obtained from the alkaline deacetylation of chitin by nitrogen, has shown interesting antibacterial and antifungal activity against a wide range of microorganisms compared to other polymers and biopolymers [16,17]. CS is a hydrophilic polyelectronic polymer that contains positive charges in an acidic medium and its properties of fluid behavior can be influenced by the molecular configuration, the number of hydrogen bonds, and the electrostatic repulsion of neighboring molecules [18]. CS has reactive functional groups susceptible to chemical modification and has been found to be a functional polymer for covalently grafting antioxidant/antimicrobial activity onto its backbone [19]. The CS polymer has a significant content of primary amines and hydroxyl groups, which gives it a high affinity for metal ions. It can be incorporated by simple chelation or ion exchange [20], making it an excellent support for layered double hydroxide (LDH) synthesis.

ZnAl consists of positively charged brucite-type layers of divalent and trivalent metal hydroxides, whose excess positive charge is compensated by anions and water molecules present in the interstitial position. Among the heterostructured nanomaterials, layered double hydroxide (LDH) was the subject of sustained attention as an inorganic component applied in many fields due to its unique physicochemical and mechanical properties, which cannot be obtained from other similar compounds [21].

Pathogenic *Escherichia coli* strains cause a variety of human diseases, which result in more than 2 million deaths each year [22,23]. With regard to *Penicillium cyclopium*, it attacks fuels and food causing their degradation. This degradation of fuel is a major loss for the economy. Knowing that the management of fuel reserves is one of the pillars of the global economy, it becomes imperative to focus on this fungus. In addition, these two types of microbes (*E. coli* and *P. cyclopium*) have developed resistance to existing antibiotics, placing them on the list of microbes requiring urgent attention.

Zinc and CS have excellent antibacterial activity [24,25]. Supra-nano hybrids materials capable of killing pathogens germs and preventing their colonization are desirable in several fields of application, such as the food industry, the textile industry, and water purification and healthcare systems. One such hybrid was prepared by Depan and Singh, 2010 [26] for application as an efficient drug carrier agent. A study of Gohi et al., 2019 [27] has also reported the preparation of a hybrid with the same chemical compounds (Chitosan, Zn, and Al) via a urea preparation method, which resulted in an effective antimicrobial compound.

In this study, the idea is to modify the method of preparation of the ZnAl/CS hybrid with respect to the preceding methods and to investigate the effects of the modification on the antimicrobial activity of the compound in a medium having a pH of about 6 and more.

In order to obtain a stable antimicrobial material, the two compounds CS and ZnAl-LDH were combined to form a hybrid material using the bi-titration method. The chemical, mechanical, and physical characteristics of the compound were determined using FTIR, thermogravimetric and differential thermal gravimetric (TGA/DTG), UV-Vis, XRD, and SEM analyses. The antimicrobial activity of the hybrid was tested against the Gram-negative bacterium *E. coli* and the fungus *P. cyclopium.* The antimicrobial synergistic effect and action mechanism of ZnAl/CS were examined. Finally, an attempt was made to optimize the parameters for the preparation of the hybrid via central composite design (CCD) of response surface methodology (RSM) tool before performing the kinetic study of the hybrid compound.

## 2. Results and Discussions

### 2.1. FT-IR Spectroscopy Analysis

**Figure 1:**
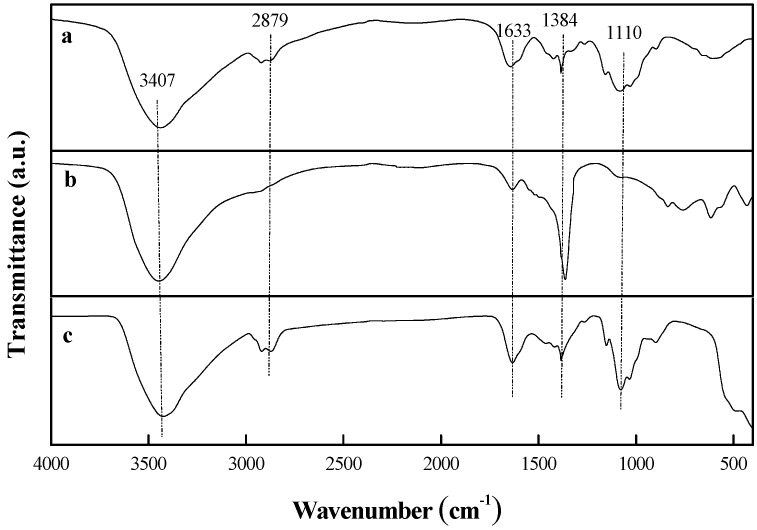
Spectra of (**a**) CS, (**b**) ZnAl-LDH, and (**c**)ZnAl/CS.

Figure 1 shows the FT-IR spectra of CS, ZnAl, and ZnAl/CS. It shows the absorption bands at 3407 cm^−1^(hydroxyl groups (*v*O–H)) in the spectra of ZnAl/CS. The bands at 3436 and 3438 cm^−1^ of CS and ZnAl, respectively, shifted to the place at 3407 cm^−1^ [28,29]. The bands at 2879 cm^−1^ were ascribed to the –CH_2_– group (aliphatic group) of CS. While there was a similar weak shoulder peak recorded at around 3000 cm^−1^ on ZnAl, it was ascribed to the OH stretching mode of interlayer water molecules hydrogen bonded to interlayer carbonate anions and appeared earlier on the hybrid ZnAl/CS at 2919 cm^−1^. This involved the birth of a bond (*v*C–H) between the hydrotalcite ZnAl and the organic compound CS [26,30,31]. The peak at around 1633 cm^−1^ on both CS and ZnAl appeared on ZnAl/CS with a more intense absorption trend, indicating the formation of the *v*CO–NH bond between the CS and ZnAl [26]. The band around 1384 cm^−1^ corresponding to C–O stretching of the primary alcoholic group and *v*_3_ (asymmetric stretching mode) of the carbonate anions showed the formation of C=OH–O–M in the hybrid ZnAl/CS [32]. An intense absorption band was located between 1000 and 500 cm^−1^, the peaks in this range could be ascribed tothe vibrational modes of different groups corresponding to N–H, M–O–M, and M–O (M = Zn or Al) [33,34].

### 2.2. Thermal Analysis (TGA/DTG)

**Figure 2:**
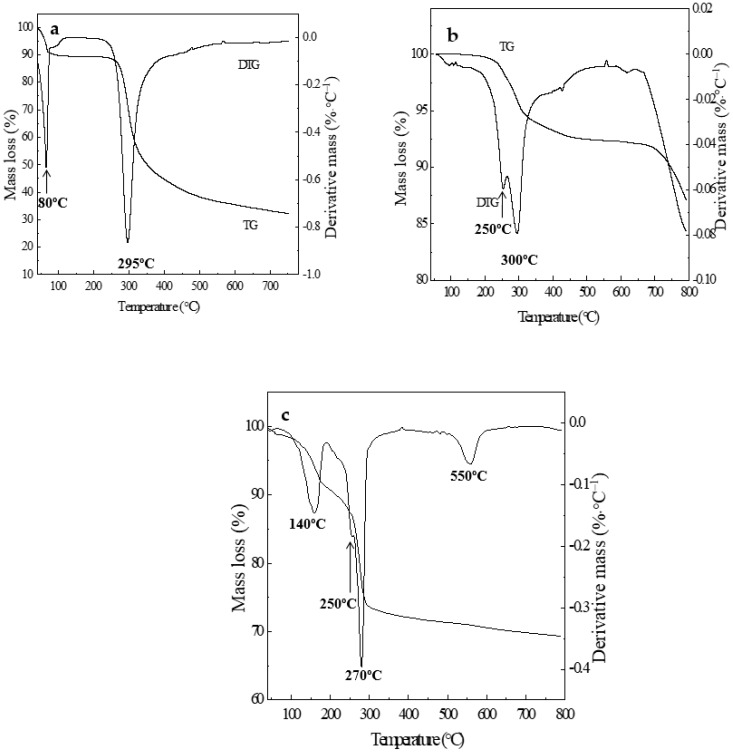
Thermogravimetric and differential thermal gravimetric (TGAand DTG) curves of (**a**) CS, (**b**) ZnAl, and (**c**) ZnAl/CS.

TGA and DTG curves of CS, ZnAl-LDH, and ZnAl/CS are shown in parts a, b, and c of Figure 2, respectively. The hydration properties of CS polysaccharides depend on primary and supra-macromolecular structure [35]. The decomposition of CS presented in two stages, the first one occurred at 47–100 °C, due to loss of water molecules with weight loss of about 9% [36]. The second stage corresponding to the primary degradation of pure CS happened at 247 °C, with a percentage weight loss of about 34%with complete degradation at 550 °C, which was similar to that reported in previous literature [35]. Generally, in CS, the decomposition process of the N-acetylated compound is overlapped by the N-deacetylated unit, thereby increasing the widening process seen at temperatures up to 400 °C [37]. The decomposition of ZnAl showed three mass loss events. The first one took place between 250 and 300 °C, corresponding to 5.5% weight loss attributed to elimination of both non-gallery surface adsorbed water and interlayer water molecules. The second event with2.5% weight loss, corresponding to the dehydroxylation and decarbonation reactions of the brucite layers [38,39], happened from 300 to 450 °C. The third mass loss of around 7%began at 650 °C and extended to reach 800 °C. This series of ZnAl mass loss resulted in a total mass loss of 15%, which was significantly lower than the mass loss of CS (43%). Three mass loss stages appeared in the TGA curve of ZnAl/CS (Table 1). The first mass loss event, corresponding to the elimination of adsorbed water at the surface and between LDH layers, was a8% weight loss completed by around 150 °C. From 150 to 250 °C (7%), there was a second weight loss ascribed to the dehydroxylation of the LDH layers and partial decomposition of the CS biopolymer. The third and last mass loss observed between 260 and 700 °C (13%) was ascribed to complete oxidative elimination of the carbonaceous residue derived from initial biopolymer degradation. Thus, ZnAl/CS recorded a total mass loss of about 28%. The results showed that the thermal stability of ZnAl was more compared with ZnAl/CS and CS and ZnAl/CS were more thermally stable than CS. The differential thermogravimetric (DTG) curves of the CS, ZnAl, and ZnAl/CS samples are also shown in Figure 2. The DTG of CS presented two endothermic peaks at 80 °C and 295 °C, while ZnAl also presented these two peaks at 250 °C and 300 °C. The maximum decomposition temperature (DTG_max_) of CS and ZnAl were recorded, respectively, as 295 °C and 300 °C, values close to those reported elsewhere for CS [40] and different for ZnAl [41,42]. The DTG curve of ZnAl/CS showed three endotherms peaks at 140 °C, 270 °C, and 550 °C with DTG_max_ at 270 °C, in addition to an edge at 250 °C.

### 2.3. UV–Vis Spectroscopy Analysis

**Figure 3:**
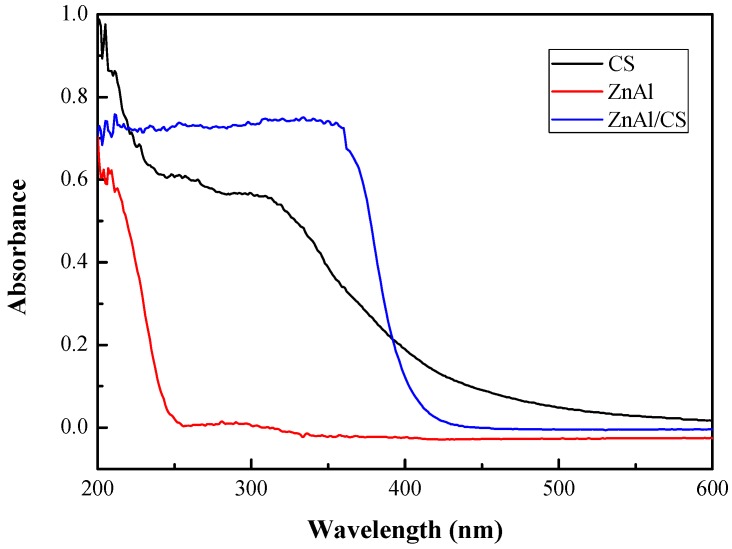
Absorbance spectra of CS, ZnAl, and ZnAl/CS.

Figure 3 shows the optical absorbance spectra for the initial CS, ZnAl-LDH, and ZnAl/CS in the range of 200–800 cm^−1^. The chitosan absorption trend was similar to the one described by Kumirska et al., 2010 [43]. Chitosan alternates far-UV chromophoric groups, N-acetylglucosamine (GlcNAc), and glucosamine (GlcN). High absorption intensity can be observed below the 220 nm wavelength and at 317 nm. The absorbance trend of ZnAl-LDH presented UV absorptions at 220 nm and 300 nm, the first one occurred at 220 nm and the second one at 300 nm. This strong absorption was attributed to the existence of nitrate anions in the interlayer galleries [44]. The ZnAl/CS spectra combined the chromophoric characteristics of the two different components, chitosan and ZnAl-LDH. From 200 to 220 nm, a mixture of the type of absorption corresponding to the type of absorption of each of the compounds was found. Also, around 300 to 360 nm, a new absorption peak was recorded; this observation suggested the existence of chemical interactions between ZnAl and CS, because absorption below 220 nm is characteristic of chitosan [43].

### 2.4. XRD Analysis

**Figure 4:**
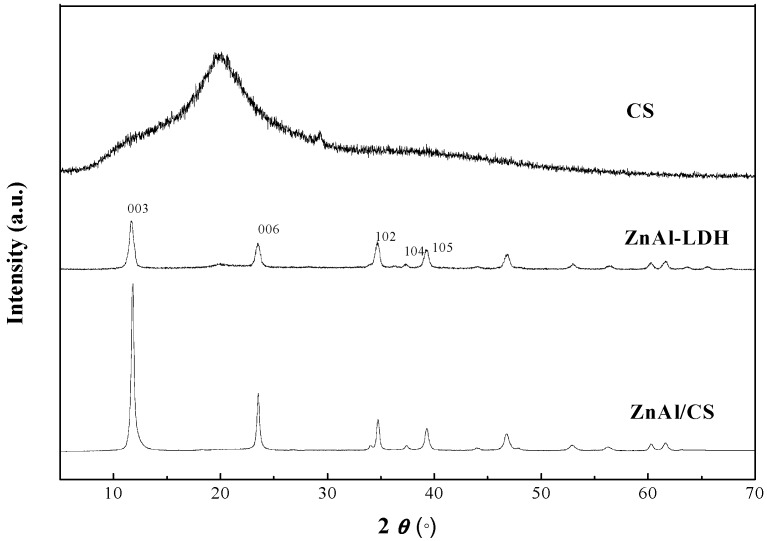
XRD patterns of CS, ZnAl-LDH, and ZnAl/CS.

The XRD patterns of CS, ZnAl, and ZnAl/CS are shown in Figure 4. The XRD pattern of CS was similar to that reported in the literature [45,46] and it exhibited two broad diffraction peaks. The location of these major peaks at 2θ = 11.4° and 20.0° was more or less close to those previously described by Bangyekan et al., 2006 [47] and Prashanth et al., 2003 [48]. This is the typical characteristic of chitosan from shrimp. The hydrotalcite-type structure was observed in the ZnAl sample after analyzing its XRD pattern. The 11.84° (003 planes) of 2θsignal, which corresponds to the interlamellar distance of the carbonated solid, was observed [49]. The d (003) characteristic peak of the hydrotalcite-like compounds was observed, where d (300) was relevant to the radius of anion in the intermediate layer and the interactions between anion and cation on the layers and represented the layer-layer spacing of LDH compounds [50]. The XRD patterns of the mixture of ZnAl/CS exhibited reflections attributed to ZnAl, showing that the addition of CS did not influence the crystalline structure of ZnAl-LDH.

### 2.5. SEM Analysis

**Figure 5:**
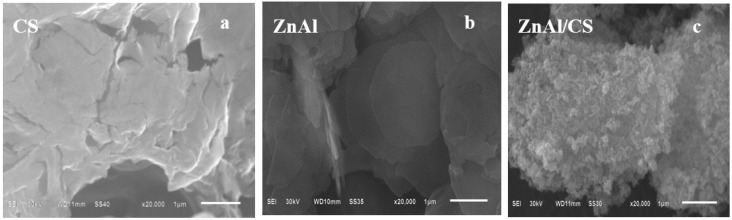
SEM images of (**a**) pure CS, (**b**) ZnAl, and (**c**) ZnAl/CS.

In Figure 5, parts a and b, respectively, reflect the compounds chitosan and layered double hydroxide ZnAl. The peculiarity observed herein was with the compound obtained by the homogenization of CS and ZnAl. Figure 5c showed that ZnAl/CS was different from the compounds constituting it, and ZnAl/CS was seen as an aggregation of CS and ZnAl-LDH. There appeared to be a crystalline deposit with the reflective characteristics of LDH, but was much smaller in size when compared with the deposit of ZnAl-LDH. To summarize, the compound obtained had all the characteristics of ZnAl/CS according to UV–Vis, FT-IR, TGA/DTG, and XRD, but differed on the supernal view of ZnAl/CS obtained by Depan and Singh, 2010 [26] and it was a bit more like ZnAl-LDH reported by Ballarin et al., 2015 [28]. This could be due to the difference in the proportion of parameters (e.g., pH, Zn/Al ratio, chitosan concentration, and NaOH amount). This indicated that the compound obtained was the hybrid ZnAl/CS. In addition, SEM images confirmed that the morphology of the ZnAl/CS hybrid was different from that of ZnAl and CS, which suggested the formation of the hybrid based on ZnAl and CS.

### 2.6. Antimicrobial Activity Study

To evaluate the antibacterial activity of CS, ZnAl-LDH, and ZnAl/CS using the agarwell diffusion method [51], *E. coli* MTCC 739 (Gram-negative bacterium) and *P. cyclopium* AS 3.4513 (spore-forming fungus) were used. Thin tablets of the sample with 1.0 cm diameter were placed into agar plates with microbial suspensions. Surprisingly, pure chitosan (CS) did not show any antibacterial activity against any of the microbes, but ZnAl and ZnAl/CS showed antibacterial activity against both *E. coli* and *P. cyclopium*. The inhibition zone surrounding the wells (in millimeters) was measured to evaluate the antibacterial activity.

**Figure 6:**
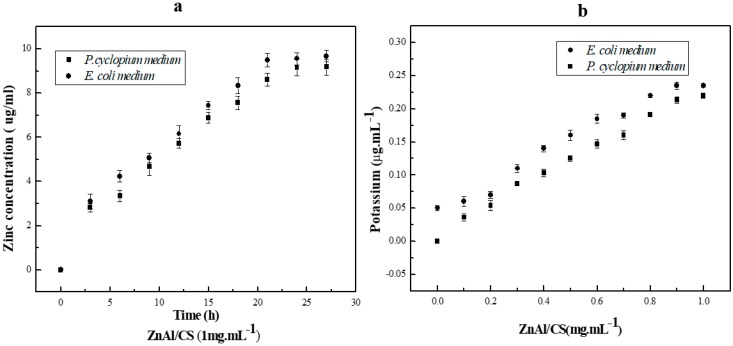
Concentration of (**a**) zinc ions and (**b**) potassium released from the ZnAl/CS hybridincell culture media (*n* = 3).

**Figure 7:**
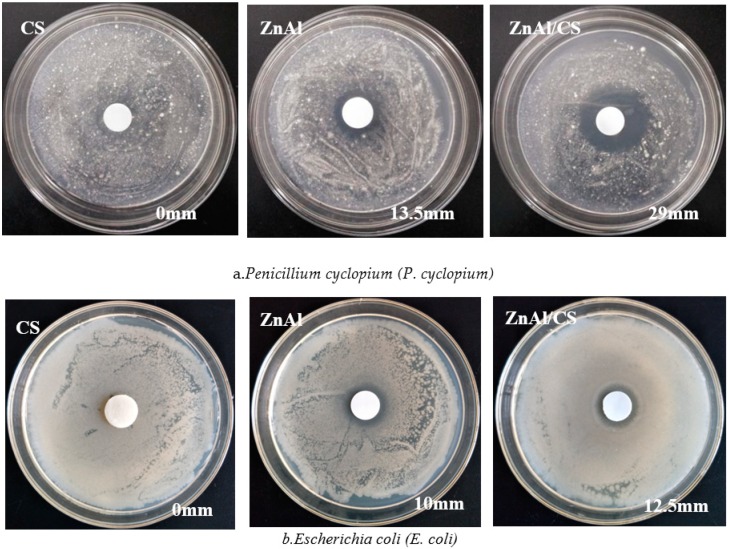
Effects of CS, ZnAl, and ZnAl/CS against (scale bar = 0.5 cm) (**a**) *P. cyclopium* and (**b**) *E. coli*.

The inhibition zone of the compounds with antimicrobial activity varied according to the organism; ZnAl had 13.5 mm of inhibition zone against *P. cyclopium* and 10 mm against *E. coli*, whereas against *P. cyclopium* and *E. coli*, ZnAl/CS produced 29 mm and 12.5 mm of inhibition zones, respectively. These results indicated that ZnAl/CS had greater antibacterial activity than ZnAl and that both compounds had prominent activity against *E. coli* compared to *P. cyclopium*. The inability of CS to inhibit the selected bacteria may be attributed either to the molecular mass of the chosen CS, which would not result in direct inhibitory activity against *E. coli* and *P. cyclopium* [52] or pH (*E. coli* pH adjusted from 7.0 to 7.4; *P. cyclopium* pH adjusted from 5.9 to 6.2) of the culture medium, which would not promote antibacterial action of CS.CS exhibits antibacterial activity only in an acidic medium because of its poor solubility above Ph 6.5 [53]. With regard to the antibacterial activity observed in ZnAl during this study, it contrasted with the observation of Ballarin et al., 2015 [28], which could be attributed to the difference in the method of manufacture of ZnAl, in the proportion of the key components as well as in the treatment of the final product, which might or might not generate certain properties. The antimicrobial activity of ZnAl hydroxide depends mostly on the status of zinc, such as the dispersion, metal surface area, and particle size. A suitable method, leading to materials with different structure, surface physicochemical and textural properties, can show better performance in antimicrobial activity. It is known that the method of preparation plays an important role in the antimicrobial performance of ZnAl hydroxides [54,55,56]. The reaction conditions of this study were as follows: zinc chloride and aluminum at a ratio of 5:2 were dissolved in 200 mL of distilled water, at a final pH of 10, under nitrogen atmosphere with magnetic stirring at 60 °C and finally, the precipitate was dried at 40 °C. The conditions of the reaction of Ballarin et al., 2015 were: nitrate salts containing Zn^2+^/Al^3+^ at 1/2 ratio with a final pH of 9.0 ± 0.2 were magnetically stirred at 21 °C and the precipitate was dried at 110 °C. Zinc has been found to possess excellent antibacterial activity [24]. Moreover, under an acidic environment (pH 4–5) of cytoplasm, LDH disintegrates into Zn and Al ions [26]. The released Zn^2+^ ions can adhere to the plasma membrane of the microbe by electrostatic attraction and then penetrate the cell membrane through the pore channel of the membrane. This deteriorates the cell membrane permeability causing leakage of intracellular ions and low-molecular-weight metabolites [57]. Several studies have already reported the release of zinc ions (Zn^2+^) as a function of time and their effects in both mammalian and microbial cell culture media [58,59,60]. However, most of these studies have not implemented a kinetic model to quantify the release of Zn^2+^ as a function of time. This could be explained by the great diversity of composites associated with zinc ions and the media difference during the preparation of the materials. However, a model was established according to the quantity of microbial colonies [61]. The model, being a rough estimate, was issued from the slopes of the curves and defined as follows: d (Log(CFU mL^−1^ 265))/d[ZnO] [61]. The Zn^2+^ ions released (Figure 6a) in the broth brought about a significant contribution to the overall antimicrobial activity of ZnO through generation of reactive oxygen species and also by direct contact with the microbial cell walls [61]. The release of zinc ions in the culture medium was not related to the size of the inhibition zone, but rather related to the reduction of microbial colonies in the liquid culture medium [61]. The inhibitory activity of ZnAl/CS, which was superior to that of ZnA1, could be attributed to activation of the inhibitory activity of CS (effective against both Gram-positive and Gram-negative bacteria [62,63]) associated with Zn^2+^ ions through ZnAl-LDH. The antibacterial activity of CS depends on different parameters. Among these parameters, the degree of deacetylation, molecular weight, solvent, and/or concentration of CS are the most important. Through these parameters, the antibacterial activity of chitosan would be obtained by the release of potassium (Figure 6b) in the culture medium [64]. This indicated that the association CS and ZnAl would trigger the inhibitory properties of CS, which would add to its own to produce this efficient antibacterial compound. This difference in the inhibitory diameter between the different types of organisms could be attributable to the difference in the membrane layer of the cell between Gram-negative *E. coli* bacterium and *P. cyclopium* fungus (Figure 7). The method of preparation is an important factor for the effectiveness of the antimicrobial activity of the hybrid. In fact, the bi-titration method is beneficial to form the heterojunction structure (ZnO, Al_2_O_3_, ZnAl_2_O_4_, Zn^2+^, and Al^3+^) compared to the previously used urea method. The heterojunction structures in the ZnAl/CS materials lead to chemical compounds and surfaces that are more or less different from those produced by the urea process. The bi-titration method produces more soluble zinc species, whereas the soluble zinc species are responsible for the full activity of ZnO [61]. This would explain why with this method ZnAl and ZnAl/CS chitosan are more effective towards the fungi *P. cyclopium* compared to against Gram-negative (*E. coli*) bacteria: the contribution of soluble zinc species was approximately 15% of the full antimicrobial activity of ZnO for Gram-negative *E. coli*, while it was almost 100% for fungi (*P. cyclopium*) [61]. The bi-titration method is more beneficial to a get high-efficiency antimicrobial agent via easy release of ion species.

### 2.7. Optimization by Response Surface Methodology

Based on previous experimental results (above) performed via “one-factor-at-a-time” experiment type, RSM was used to optimize the preparation conditions and obtain sufficient antibacterial activity from the selected materials. The selected effective variables were pH (P), ratio ZnAl (R), andCS concentration (C, g/L). The experimental design suggested by central composite design (CCD) was applied because it helps to optimize effective parameters and analyze interactions between parameters and also produces results within a minimum of number of experiments. The bacterium chosen to conduct this part of the study was *E. coli*.

#### 2.7.1. Data Analysis and Evaluation of CCD Model

The antibacterial effectiveness experiment was conducted using the CCD design, and the results are presented in Table 1. The results of the experiment showed that each factor had a kind of influence on the final compound affecting its antibacterial efficacy against *E. coli,* with different sizes of inhibition zones. A statistical analysis of variance (ANOVA) was conducted by using a software to investigate not only the suitability and relevance of the model, but also the effects of individual variables on interaction effects on the response (zone of inhibition, mm). ANOVA showed (Table 2) that a quadratic model best fitted to explain the functionality of the system. This was confirmed by a coefficient of determination R^2^ value of 0.995, an F-test value of 239.77, and a statistically significant probability value ($P_{\mathrm{model}}$ > F (< 0.0001)). The goodness of the model was also shown by a non-significant lack of fit $(P_{\mathrm{model}}$ > F = 0.3493) and a predicted significant R^2^ value and an adjusted R^2^ value of 0.975 and 0.991, respectively. Adequate precision that measured the signal-to-noise ratio value of 45.211 was greater than four and demonstrated an adequate signal. The relatively lower value of the coefficient of variation (CV 3.66%) indicated the good precision and reliability of the experiments [65] as well as confirmed the fitness of the quadratic model.

##### Main Effects of the Independent Variables P (Initial pH), R (Ratio ZnAl), and C (CS Concentration (g/L)) and Interactions between Parameters

When considering the independent variables individually, CCD study indicated that each parameter had a determinant role in the formation and inhibition efficiency of the antibacterial compound ZnAl/CS. Indeed, P strongly affected the formation of ZnAl/CS, first through the dissolution of CS which took place in an acidic medium, and finally through the connection between ZnAl and CS which occurred in an alkaline medium. A lack of control about this factor P, led to a modification and/or stoppage in the formation of ZnAl/CS, inducing a final compound inefficiency through a rather poor inhibition zone. Regarding R, a ratio lower or superior to 3 influenced the width of the zone of inhibition differently. With R < 3, ZnAl/CS produced a very small inhibition zone. At R > 3, the inhibition zone was enlarged, while remaining largely lower than the ZnAl/CS zone when R = 3. C represented the perfect dosage of CS at which ZnAl should be associated to produce a compound with a more effective antibacterial capacity. According to the experiment, the zone of inhibition of ZnAl was lower than that of ZnAl/CS, which showed the importance of controlling C. In terms of the significant coefficients, the independent variables P, R, and C were all highly significant with *p* ≤ 0.0001.

In terms of interaction, PC and RC with *p*-values< 0.05 were both significant terms for influencing the inhibition ability (inhibition zone ZI) of ZnAl/CS. It was quite remarkable that all of the significant interaction terms contained the independent variable C, implying that C played a key role in the process of optimization or improvement of ZnAl/CS inhibition ability. The quadratic terms P^2^, R^2^, and C^2^ were all highly significant (*p* < 0.0001). After analyzing the independent and dependent variables, we obtained a regression equation that could predict the response under a given range. The linear regression equation was obtained, after elimination of insignificant terms to improve the regression model for the responses (zone of inhibition). The quadratic model Equation (1) in terms of the coded factors was given as follows:

ZI = +16.60 + 1.65P + 2.05R + 0.69C + 0.43PC + 0.55RC − 2.97P^2^ − 3.58R^2^ − 0.77C^2^ (1)

**Figure 8:**
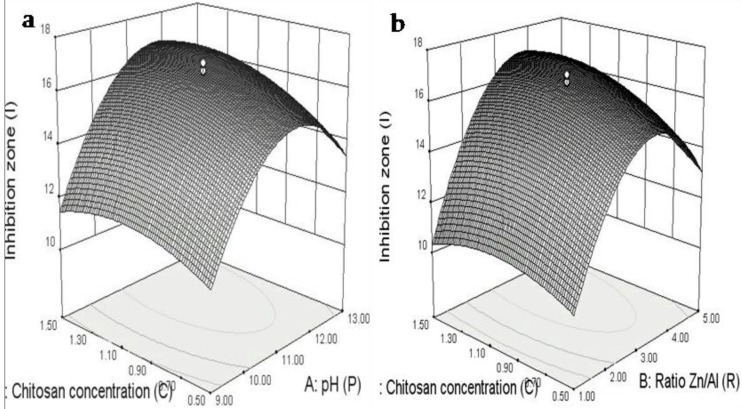
Surface of zone of inhibition (ZI) versus P and C (**a**) R and C (**b**).

To study the interaction between parameters in depth, the quadratic model’s regression equation graphical representation was used. The three-dimensional response surface plot in question was also used to determine the possible optimum conditions of each factor in order to maximize the ZnAl/CS inhibition capacity. The model confirmed the existence of significant particular interactions principally between C and the two other terms P and R. We further characterized the interaction in the range of the process variables. Figure 8a,b shows contour curves and iso-response surface for the optimization of the three independent variables. The combined effect on the inhibition zone of two out of three independent variables was plotted, while the third variable was kept at the zero level. The elliptical nature of the contour plot of Figure 8a,b between P/C and R/C indicated that significant interaction between the variables P, R, and C had an important effect on the inhibition zone (ZI). Figure 8a depicts the response surface described by the model equation to estimate the inhibition zone that explains antibacterial activity over independent variables: P and C with the actual factor R at 3. According to the model graph, it can be interpreted that a maximum inhibition zone of 17 mm was obtained by conducting P at 11 with C at 1 g/L. Increasing or decreasing both P and ratio of C will significantly reduce the diameter (zone) of inhibition. Figure 8b represents the combined effect of R and C on inhibition zone Y, while the third variable P was held at the zero level. The elliptical nature of the contour plot between R and C indicated that significant interaction between these two variables had an effect on ZI of ZnAl/CS. The experimental versus model predicted values for Y of ZnAl/CS based on computation of the linear correction coefficient confirmed a reasonable agreement between the experiment and model.

#### 2.7.2. Optimization Analysis

**Figure 9:**
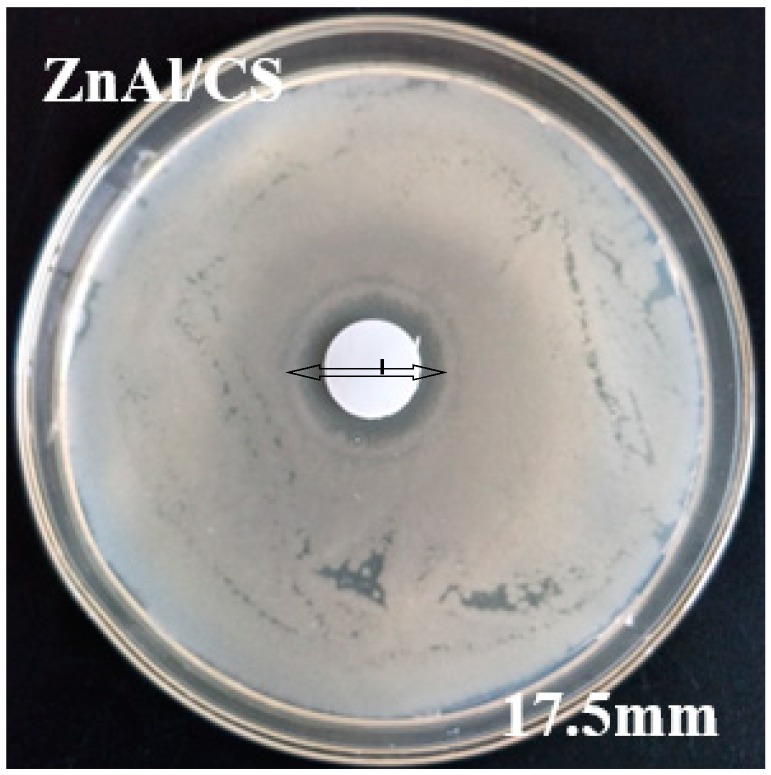
Effects of optimized ZnAl/CS (ZnAl/CSo) against *E. coli* (scale bar = 0.5 cm).

**Figure 10:**
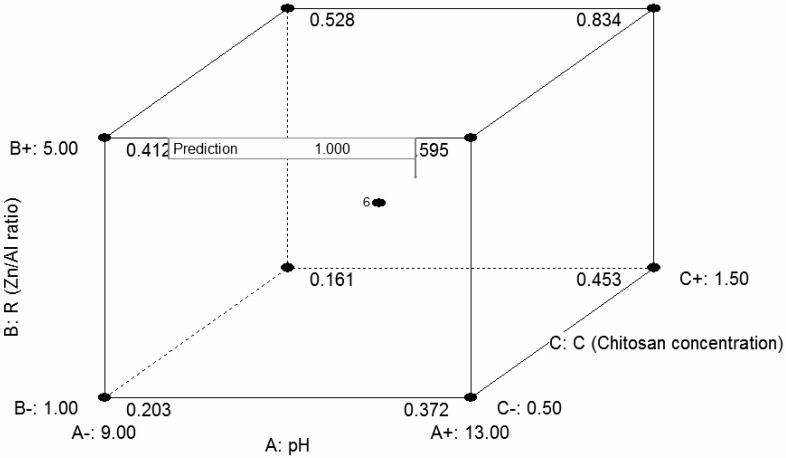
Cube of optimization of ZnAl/CS preparation.

After analyzing the influencing variables and the appropriate model, the next step was to accurately determine the corresponding value of each important parameter individually or in a set that can improve the antibacterial inhibitory capacity of ZnAl/CS and to achieve a zone of inhibition broader (larger) than the previous one. This optimization was done via RSM, by applying a canonical analysis which is one of the multivariate linear statistical analyses used to locate the stationary point of response surface and to determine whether it represents a maximum, minimum, or saddle point. P: 11.27, R: 3.27, and C: 1.07 g/L were the optimal conditions for obtaining the widest zone of inhibition as predicted by the model. The theoretical inhibition zone predicted using the above conditions was 17.07 mm. In order to validate the applicability of the model, a series of experiments was carried out using the optimal parameter values suggested by the conical analysis, P: 11.27, R: 3.27, and C: 1.07 g/L. As a result, a relatively higher experimental value, but close to that predicted by the model, was obtained, see Figure 9 (17.3 ± 0.65 mm). This satisfactory result confirmed, in addition to the model predicted, the effectiveness of the modeling tool RSM. Figure 10 summarizes the optimization model.

One of the most striking differences between this study and our previous study [29] was the antibacterial activity of the ZnAl/CS hybrid against *P. cyclopium* and *E. coli*. The ZnAl/CS hybrid was more effective against *P. cyclopium* fungi with a zone of antimicrobial inhibition of 29 ± 0.7 mm (bi-titration) against 22.3 ± 0.5 mm (urea method of preparation) and less effective against *E. coli* compared to the previous study with a zone of inhibition of 17.3 ± 0.65 mm (bi-titration preparation method) against 24.2 ± 0.8 mm (urea method of preparation).

The urea method of preparation is a high crystallinity method with more complete bonds leading to firmly attached components [66]. This leads to a slower and lower release of ions contained in the material [67]. This has the consequence of causing a weak antimicrobial activity and a rapid propagation of microbes over time. The urea preparation method thus leads to an antimicrobial activity moderately effective towards the fungus.

In contrast to the urea method of preparation, the bi-titration preparation method produces weak crystallinity but better heat stability material [68], in addition to showing a high rate capability and impressive specific capacity and cycle property [69]. This compound structure will produce a rapid dispersion of the ions contained in the material and an immediate effect on the environment. This will result in a permanent and effective contact of the ions with the microbes which will cause their inhibition.

### 2.8. Antibacterial Killing Kinetic Assays of ZnAl/CS

Since the *E. coli* growth cycle extends over a period of 24 h, we set the duration of the experiment to a growth cycle, that was why the incubation time was 24 h. This study was carried out in a liquid medium, so it was possible to deduce a kinetic model showing the amount of potassium released as a function of the microbial colony reduction rate. Also, potassium release by *E. coli* has already been studied and a kinetic model has already been established and described in the form of: 10 μM K^+^/min for 0.5 mg/mL of propyl paraben. This previous study was able to relate the variation in the size of the inhibition zone as a function of the quantity of potassium released without establishing an equational model [70].

**Figure 11:**
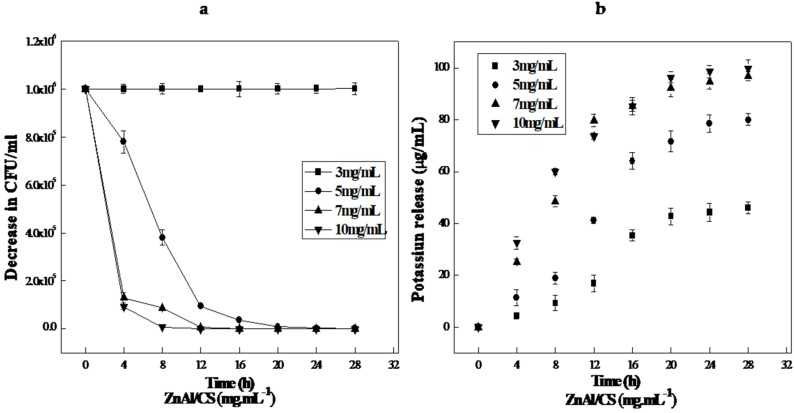
Response of *E. coli* to ZnAl/CS in Müller–Hinton medium. Filter-sterilized Mueller-Hinton broth MHB* medium was supplemented with various concentrations of ZnAl/CS and incubated for 24 h at 37 °C under anaerobic conditions. The OD_600_ of each culture was measured. (**a**) *E. coli* killing and (**b**) Potassium release curves

A kinetic time-kill study was performed at different concentrations of ZnA1/CS against *E. coli*. The optical density at 600 nm (OD_600_) was used to measure and control bacterial growth. All the results confirmed the antibacterial efficacy of ZnA1/CS. This was further confirmed, when beyond the addition of 8 mg/mL of ZnAl/CS, the optical density no longer varied as a function of time, which indicated the total inhibition of bacterial growth (Figure 11). Here, the evolution of cell growth was different from some studies, and this would be due to the difference between the growth media. Average cell mass is known to vary strongly with the growth medium, being greater in media that support rapid growth [71]. Through the transparency of the culture suspensions, the minimum inhibitory concentration (MIC) measured was 3 mg/mL and the minimum bactericidal concentration (MBC) after 24 h of *E. coli* culture suspensions was 7 mg/mL.

## 3. Materials and Methods

### 3.1. Materials

Chitosan from shrimp shells (Mw 1.5 × 10^5^ and ≥91% deacetylate) was purchased from Sinopharm Chemical Reagent Co., Ltd. (Shanghai, China). AlCl_3_.6H_2_O (>97%) and KOH were obtained from Heng Xing Chemical Preparation Co., Ltd (Tianjin, China). ZnCl_2_ (98%) was purchased from Fuchen Chemical Reagent Factory Co., Ltd (Tianjin, China). NaOH (≥96%) was procured from Xilong Chemical Co., Ltd (Guangdong, China). Lactic acid (Mw 90.07) was purchased Shanghai Meryer Chemical Technology Co., Ltd (Shanghai, China).

### 3.2. Preparation of ZnAl-LDH and ZnAl/CS

The ZnAl/CS hybrid was prepared using a co-precipitation technique proposed by Depan and Singh, 2010 [26] with slight modifications. 0.5 g of CS of low acetylation degree was dissolved in an aqueous solution of lactic acid (2 mL) and 8.5 g of NaOH was added to a 300 mL biopolymer solution to reach a pH value of 10 to ensure the complete deprotonation of carboxylic groups of CS. Aqueous solutions of zinc and aluminum chloride were prepared from chemicals of analytical grade. Zn/Al at a ratio of 5/2 was dissolved in 200 mL of decarbonated bi-distilled water and added dropwise to the biopolymer solution under nitrogen atmosphere to avoid ${\mathrm{CO}}_{3}^{2-}$ contamination; the whole mixture was magnetically stirred vigorously at 60 °C. The aqueous solutions of Zn and Al were continuously added at a rate of 20 mL/h. The precipitate was aged in the mother liquid for 24 h, the white solid product was isolated by repeated centrifugation, and washed with decarbonized water, then finally dried at 40 °C. Virgin ZnAl-LDH without biopolymer, denoted as ZnAl, was synthesized following the same procedure, however, the biopolymer CS was replaced by a bi-distilled water solution. During the centrifugation, sealed containers were used to avoid absorption of atmospheric carbon dioxide. The deionized bi-distilled water was used for the synthesis and washing steps, but was first decarbonized by boiling. Subsequent cooling of the prepared solution in the absence of CO_2_ was carried out by means of a gas-washing bottle filled with a KOH solution. All solutions were prepared with deionized water (resistivity of 18.2 mX cm) which was obtained with a Millipore ultra-pure water system, previously distilled and decarbonized by boiling plus bubbling N_2_. The compounds studied in this study were labeled as CS, ZnAl, and ZnAl/CS.

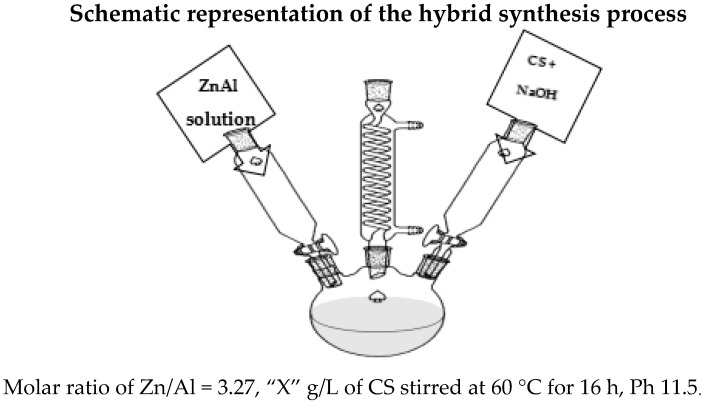



### 3.3. FT-IR Spectroscopy Analysis

The FT-IR spectra of CS, ZnAl, and ZnAl/CS samples were obtained with Perkin-Elmer Spectrum GX in the wave number ranging from 4000 to 400 cm^−1^. Powder samples were molded in potassium bromide KBr pellets and analyzed at a resolution of 2 cm^−1^.

### 3.4. Thermal Analysis (TGA/DTG)

Thermogravimetric and differential thermal gravimetric analyses (TGA/DTG) were performed on a Perkin-Elmer TGA-7thermal analyzer in a temperature range of 50 to 900 °C. 10 mg of each composite sample was loaded in an open ceramic crucible, and heated under nitrogen atmosphere at a heating rate of 20 °C min^−1^.

### 3.5. UV-vis Spectroscopy Analysis

Measurements of the UV–vis spectra at room temperature for CS, ZnAl, and ZnAl/CS solids were recorded on a SHIMADZU UV-2550 spectrophotometer (Kyoto, Japan). In order to record the measurements, a solution of chitosan (10^−2^ g/L) was prepared by adding a stoichiometric amount of 10^−1^ M perchloric acid to the chitosan solution. Given the water content and the degree of deacetylation of the chitosan, the chitosan–perchloric acid solution was then stirred at an appropriate speed to complete the dissolution before recording the measurements. Samples of ZnAl and ZnAl/CS were prepared by depositing the aqueous dispersions onto quartz glass slides and drying them in air. The concentration of ZnAl and the ratio of ZnAl to ZnAl/CS were the same for both the UV-Vis spectra.

### 3.6. XRD Analysis

The wide angle X-ray diffractometer patterns were collected on a Rigaku (Tokyo, Japan) D/max-2550PC, with Cu-*K*a radiation of 15,406 Å and an operating voltage at 50 kV. The scanning was in the 2θ range of 2° to 70° with a scan speed of 5°/min.

### 3.7. SEM Analysis

Scanning electron micrographs (SEM) were obtained through a JEOL JSM-6700F instrument (Tokyo, Japan). The samples (CS, ZnAl, and ZnAl/CS solids) were spread on a double-sided conducting adhesive tape that was pasted to a metallic stub, coated with gold (100 µg), and then observed atan accelerating voltage of 20 kV.

### 3.8. Organism and Culture Conditions

*E. coli* MTCC 739 and *P. cyclopium* AS 3.4513 were kept at the Xiangtan University General Microbiological Culture Collection Center. The fungus *P. cyclopium* strain was incubated on potato dextrose (PD) agar medium at 23 °C for 3 days before dilution and inoculation, while *E. coli,* the Gram negative strain, was maintained in mineral salt (MS) media, pH 7.0, at 37 °C for 24 h before multiple dilution and use.

#### In Vitro Antibacterial Activity

The antibacterial efficiency of ZnAl/CS was evaluated through determining the antibacterial susceptibility, by the agar well diffusion method, of bacteria after exposure to the tested samples. Gram-negative *E. coli* bacteria and *P. cyclopium* fungi were used as target organisms. Müller–Hinton broth (MHB) (Canspec Scientific Instruments Co., Ltd. Merck, KGaA, Shanghai, China) was chosen to prepare the microbial suspension; the turbidity of the suspension was balanced with 0.5 McFarland standard. The broth plates were inoculated with the microorganisms (bacteria and fungi) and slightly perforated with a glass capillary to create wells that were filled with 1.0 cm diameter of CS, ZnAl, and ZnAl/CS. The pills (CS, ZnAl, ZnAl/CS) were irradiated under a UV lamp for 30 min to eliminate microbial contamination. Each sample was added into a 10 mL Luria-Bertani LB liquid culture medium which was then inoculated with the microbial suspension. All antimicrobial experiments involving both cultures were carried out in sterile 250 mL shake flasks or 90 mm agar plates. Indeed, 100 mL cultures of *P. cyclopium* and *E. coli* strains were partially transferred onto PD and MS media in order to perform the antimicrobial tests. The plates were incubated for 24 h at 37 °C for *E. Coli* and 72 h at 28 °C for *P. cyclopium*. Finally, the inhibition zone surrounding the wells (in millimeters) was measured to evaluate antibacterial activity.

### 3.9. Optimization of ZnAl/CS Hybrid Preparation Conditions

The response surface methodology (RSM) is a powerful statistical tool. It relies on quantitative data from an accurate fact-finding conception to simultaneously determine and solve multivariate equations. Its advantage is the reduction of the number of experimental tests necessary to evaluate the impact of several parameters and the effect of their interactions. After deducting the best experimental conditions from the “one-factor-at-a-time” experimental method, RSM was applied to determine the effect of initial pH, ZnAl ratio, and CS concentration on the bacterial inhibition efficiency of the hybrid ZnAl/CS. The CCD tool of RSM was chosen for the experimental test with the three independent variables that were the initial pH (P), ZnAl (R) ratio, and chitosan concentration (C, g/L), while optimizing the zone of inhibition (ZI) as response variable (Y) of the antibacterial study. Each independent variable was coded at three levels between −1 and +1, while the variables initial pH (P), ZnAl ratio (R), and chitosan concentration (C, g/L) were changed in the ranges shown in Table 3. A set of 20 experiments was achieved with three replications at the design center to evaluate the pure error. The experiments were performed in a random order, as required by many design procedures. After reaction, response ZI (Y) was measured and the statistical software package Design Expert (version 8.0.6) was applied for regression analysis of the trial data and to plot response surface. The model generated during the implementation of the RSM was validated by performing experiments on a given optimal parameter. The polynomial model of the first-order equation applied to predict the response variable ZI (Y) for the case of three independent variables is expressed as follows (2):

(2) $$Y=\beta_{0}+\overset{k}{\underset{i=1}{\sum }}\beta_{i}X_{i}+\overset{k}{\underset{i=1}{\sum }}\beta_{\mathrm{ii}}X_{i}^{2}+\overset{k}{\underset{i<j}{\sum }}\beta_{\mathrm{ij}}X_{i}X_{j}$$

where Y is the predicted response, $\beta_{0}$ is a constant, $\beta_{i}$ is the ith linear coefficient, $\beta_{\mathrm{ii}}$ is the ith quadratic coefficient, $X_{i}{\;\mathrm{and}\;X}_{j}$ are the coded values of independent variables, the terms $X_{i}X_{j}$ and $X_{i}^{2}$ represent the interaction and quadratic terms, respectively, and k is the number of independent variables.

### 3.10. Kinetic Study

An antibacterial kinetic test was performed, and *E. coli* was selected for the kinetic study as the index bacterium. The culture was incubated overnight at 37 °C. The bacterial suspension (10^6^–10^7^ CFU/mL) was treated with 1,3, 5, 7, and 10 mg/mL of CS, ZnAl, and ZnAl/CS hybrid. The samples were incubated at 37 °C in an aerobiosis and the bacterial growth rates were assessed by monitoring optical density at 600 nm (OD_600_) at regular time intervals using a UV–Vis spectrophotometer while using normal saline as a control.

### 3.11. Statistical Analysis

Each sample was analyzed individually in triplicate and the results were expressed as the mean value (*n* = 3) of the standard error of mean. Response surface methodology (RSM) was employed to build the best model and optimize the antibacterial compound system using Design Expert version 8.0.5.0 (Stat Ease Inc. USA). One-way ANOVA for response surface model was carried out to assess the main effects of each parameter and its interactions, and the accuracy and quality of the fitted model.

## 4. Conclusions

Both methods used for the preparation of the ZnAl/CS hybrid in previous and current studies resulted in the synthesis of an antimicrobial compound. However, the hybrid had more or less different characteristics according to the preparation methods. In this study, ZnAl/CS exhibited a potent and prolonged antibacterial activity against *P. cyclopium*, whereas in the previous study its action was moderately effective against the fungi. This could be attributed to the use of the bi-titration method, which is beneficial to form heterojunction structures (ZnO, Al_2_O_3_, ZnAl_2_O_4_, Zn^2+^, Al^3+^) with weak crystallinity, promoting an easy and fast dispersion of metal ions in the culture medium. Furthermore, improvement of antibacterial activity against *E. coli* by optimization via CCD of RSM was successful with a 41.6% widening of the inhibition zone. Finally, ZnAl/CS retained antimicrobial activity in a culture medium at pH greater than or equal to 6 with an antimicrobial activity that could be directly improved. ZnAl/CS appears to be a promising antimicrobial agent, which could be useful in many areas.

## Figures and Tables

**Table 1:** Experimental central composite design matrix and its response and predicted value.

Run	Experimental Variables			Response Y (mm)	
	P	R	C(g/L)	Expt.	Predicted
1	11.00	3.00	1.00	16.7	16.60
2	9.00	1.00	0.50	5.9	5.91
3	11.00	3.00	1.00	17	16.60
4	11.00	3.00	1.00	16.5	16.60
5	11.00	3.00	1.84	15.8	15.57
6	9.00	5.00	1.50	10.3	10.44
7	11.00	−0.36	1.00	3.1	3.02
8	13.00	5.00	1.50	14.6	14.69
9	13.00	1.00	0.50	8.3	8.27
10	13.00	5.00	0.50	11	11.37
11	7.64	3.00	1.00	5.1	5.44
12	11.00	3.00	0.16	13.2	13.26
13	11.00	6.36	1.00	10	9.91
14	9.00	5.00	0.50	9.2	8.82
15	11.00	3.00	1.00	16.4	16.60
16	11.00	3.00	1.00	16	16.60
17	14.36	3.00	1.00	11.5	10.99
18	9.00	1.00	1.50	5.6	5.34
19	13.00	1.00	1.50	8.9	9.39
20	11.00	3.00	1.00	17	16.60

**Table 2:** Analysis of variance ANOVA for the response surface quadratic model (a = 0.05).

Source	Sum of Squares	DF	Mean Square	F	*p*-Value
Model	389.68	9	43.30	239.77	<0.0001
P	37.28	1	37.28	206.44	<0.0001
R	57.42	1	57.42	318.00	<0.0001
C	6.43	1	6.43	35.62	0.0001
PR	0.020	1	0.020	0.11	0.7462
PC	1.44	1	1.44	8.00	0.0179
RC	2.42	1	2.42	13.40	0.0044
P^2^	126.70	1	126.70	701.61	<0.0001
R^2^	185.09	1	185.09	1024.96	<0.0001
C^2^	8.61	1	8.61	47.69	<0.0001
Residual	1.81	10	0.18		
Lack of Fit	1.07	5	0.21	1.44	0.3493
Pure Error	0.74	5	0.15		
Core Total	391.49	19			
R^2^	0.9954				
Adjusted R^2^	0.9912				
Predicted R^2^	0.9753				
Adequate precision	45.211				
CV	3.66				

**Table 3:** Experimental range and levels of the independent variables.

Independent Variables	Symbols	Units	Code Levels		
			−1	0	+1
pH	P		9	11	13
Ratio Zn/Al	R		1	3	5
Chitosan concentration	C	g/L	0.50	1.00	1.50

## References

[1] Sharaha U., Rodriguez-Diaz E., Riesenberg K., Bigio I.J., Huleihel M., Salman A. (2017). Using Infrared Spectroscopy and Multivariate Analysis to Detect Antibiotics’ Resistant *Escherichia coli* Bacteria. Anal. Chem..

[2] Belete T.M. (2019). Novel targets to develop new antibacterial agents and novel alternatives to antibacterial agents. Hum. Microbiome J..

[3] Palla-Rubio B., Araújo-Gomes N., Fernández-Gutiérrez M., Rojo L., Suay J., Gurruchaga M., Goñi I. (2019). Synthesis and characterization of silica-chitosan hybrid materials as antibacterial coatings for titanium implants. Carbohydr. Polym..

[4] Mulongo-Masamba R., Hamidi A.E., Bouyahya A., Halim M., Arsalane S. (2018). A novel hybrid β-chitin/calcium phosphate functionalized with copper nanoparticles for antibacterial applications. J. Environ. Chem. Eng..

[5] Patil P.P., Bohara R.A., Meshram J.V., Nanaware S.G., Pawar S.H. (2019). Hybrid chitosan-ZnO nanoparticles coated with a sonochemical technique on silk fibroin-PVA composite film: A synergistic antibacterial activity. Int. J. Biol. Macromol..

[6] Rokesh K., Sakar M. (2019). Trong-OnDo, 2-(Aminomethyl pyridine)SbI_5_: An emerging visible-light driven organic-inorganic hybrid perovskite for photoelectrochemical and photocatalytic applications. Mater. Lett..

[7] Zhou D., Wang Y., Zhu J., Yu J., Hu Z. (2019). Mechanically strong and highly efficient healable organic/inorganic hybrid dynamic network. Polymer.

[8] Manchand H., Mannari V. (2019). Super photo-base initiated organic-inorganic hybrid coatings by plural-cure mechanisms. Prog. Org. Coat..

[9] Cunha V.R.R., Guilherme V.A., De Paula E., De Araujo D.R., Silva R.O., Medeiros J.V.R., Leite J.R.S.A., Peterson P.A.D., Foldvari M., Petrilli H.M. (2016). Delivery system for mefenamic acid based on the nanocarrier layered double hydroxide: Physicochemical characterization and evaluation of anti-inflammatory and antinociceptive potential. Mater. Sci. Eng. C.

[10] Yuab S., Xu X., Feng J., Liu M., Hu K. (2019). Chitosan and chitosan coating nanoparticles for the treatment of brain disease. Int. J. Pharm..

[11] Vakili M., Rafatullah M., Salamatinia B., Abdullah A.Z., Ibrahim M.H., Tan K.B., Gholami Z., Amouzgar P. (2014). Application of chitosan and its derivatives as adsorbents for dye removal from water and wastewater: A review. Carbohyd. Polym..

[12] Omidi S., Kakanejadifard A. (2019). Modification of chitosan and chitosan nanoparticle by long chain pyridinium compounds: Synthesis, characterization, antibacterial, and antioxidant activities. Carbohydr. Polym..

[13] Wang X.J., Lou T., Zhao W.H., Song G.J. (2016). Preparation of pure chitosan film using ternary solvents and its super absorbency. Carbohydr. Polym..

[14] Ge H.C., Hua T.T. (2016). Synthesis and characterization of poly (maleic acid)-grafted crosslinked chitosan nanomaterial with high uptake and selectivity for Hg (II) sorption. Carbohydr. Polym..

[15] Zubair M., Daud M., McKay G., Shehzad F., Al-Harthi M.A. (2017). Recent progress in layered double hydroxides (LDH)-containing hybrids as adsorbents for water remediation. Appl. Clay Sci..

[16] Verlee A., Mincke S., Stevens C.V. (2017). Review: Recent developments in antibacterial and antifungal chitosan and its derivatives. Carbohydr. Polym..

[17] Thomas V., Yallapu M.M., Sreedhar B., Bajpai S.K. (2009). Fabrication, characterization of chitosan/nanosilver film and its potential antibacterial application. J. Biomater. Sci. Polym. E.

[18] Chien R.C., Yen M.T., Mau J.L. (2016). Antimicrobial and antitumor activities of chitosan from shiitake stipes, compared to commercial chitosan from crab shells. Carbohydr. Polym..

[19] Choi C., Nam J.P., Nah J.W. (2016). Application of chitosan and chitosan derivatives as biomaterials. J. Ind. Eng. Chem..

[20] Murugadoss A., Chattopadhyay A. (2008). A “green” chitosan-silver nanoparticle composite as a heterogeneous as well as micro-heterogeneous catalyst. Nanotechnology.

[21] Ruiz-Hitzky E., Aranda P., Darder M., Rytwo G. (2010). Hybrid materials based on clays for environmental and biomedical applications. J. Mater. Chem..

[22] FAO (2011). Food Chain Crisis Management Framework. Preventing *E. coli* in Food.

[23] Kaper J.B., Nataro J.P., Mobley H.L.T. (2004). Pathogenic *Escherichia coli*. Nat. Rev. Microbiol..

[24] Mishra G., Dash B., Pandey S., Mohanty P.P. (2013). Antibacterial actions of silver nanoparticles incorporated Zn-Al layered double hydroxide and its spinel. J. Environ. Chem. Eng..

[25] Li B., Zhang Y., Yang Y., Qiu W., Wang X.X., Liu B.P., Wang Y.L., Sun G.C. (2016). Synthesis, characterization, and antibacterial activity of chitosan/TiO_2_ nanocomposite against *Xanthomonas oryzae pv. oryzae*. Carbohydr. Polym..

[26] Depan D., Singh R.P. (2010). Preparation and Characterization of Novel Hybrid of Bio-Assisted Mineralized Zn-Al Layered Double Hydroxides Using Chitosan as a Template. J. Appl. Polym. Sci..

[27] Gohi B.F.C.A., Zeng H.Y., Cao X.J., Zou K.M., Shuai W., Diao Y. (2019). Preparation of the Hybrids of Hydrotalcites and Chitosan by Urea Method and Their Antimicrobial Activities. Polymers.

[28] Ballarin B., Mignani A., Mogavero F., Gabbanini S., Morigi M. (2015). Hybrid material based on ZnAl hydrotalcite and silver nanoparticles for deodorant formulation. Appl. Clay Sci..

[29] Gohi B., Zeng H.Y., Pan A.D. (2016). Optimization and Characterization of Chitosan Enzymolysis by Pepsin. Bioengineering.

[30] Valentin R., Bonelli B., Garrone E., Renzo D.F., Quignard F.O. (2007). Accessibility of the Functional Groups of Chitosan Aerogel Probed by FT-IR-Monitored Deuteration. Biomacromolecules.

[31] Saber O., Tagaya H. (2003). Preparation and Intercalation Reactions of Zn-Sn LDH and Zn-Al-Sn LDH. J. Porous Mater..

[32] Sifontes A.B., Gonzalez G., Ochoa J.L., Tovar L.M., Zoltan T., Cañizales E. (2011). Chitosan as template for the synthesis of ceria nanoparticles. Mater. Res. Bull..

[33] Chen Y.F., Zhou S.H., Li F., Chen Y.W. (2010). Synthesis and photoluminescence of Eu-doped Zn/Al layered double hydroxides. J. Mater. Sci..

[34] Braga T.P., Longhinotti E., Pinheiro A.N., Valentini A. (2009). Synthesis of hybrid spheres for the dehydrogenation of ethylbenzene in the presence of CO_2_. Appl. Catal. A Gen..

[35] Cardenas G., Miranda S.P. (2004). FTIR and TGA Studies of chitosan composite films. J. Chil. Chem. Soc..

[36] Kumar S., Koh J. (2012). Physiochemical, optical and biological activity of chitosan-chromone derivative for biomedical applications. Int. J. Mol. Sci..

[37] Taboada E. (2003). Retención de Metales Pesados Utilizando Quitosano y Derivados. Ph.D. Thesis.

[38] Hajibeygi M., Shabanian M., Khonakdar H.A. (2015). Zn-AL LDH reinforced nanocomposites based on new polyamide containing imide group: From synthesis to properties. Appl. Clay Sci..

[39] Chen Y.F., Wang X.Q., Luo S.D., Bao Y. (2016). Synthesis of new Tb-doped Zn-Al LDH/tryptophan hybrids and their fluorescent property. J. Rare Earth..

[40] Ahuja M., Bhatt D.C. (2018). Polyelectrolyte complex of carboxymethyl gum katira-chitosan: Preparation and characterization. Int. J. Biol. Macromol..

[41] Elhalil A., Elmoubarki R., Machrouhia A., Sadiq M., Abdennouri M., Qourzal S., Barka N. (2017). Photocatalytic degradation of caffeine by ZnO-ZnAl_2_O_4_ nanoparticles derived from LDH structure. J. Environ. Chem. Eng..

[42] Ahmed A.A.A., Talib Z.A., Hussein M.Z.B. (2012). Thermal, optical and dielectric properties of Zn-Al layered double hydroxide. Appl. Clay Sci..

[43] Kumirska J., Czerwicka M., Kaczyński Z., Bychowska A., Brzozowski K., Thöming J., Stepnowski P. (2010). Application of Spectroscopic Methods for Structural Analysis of Chitin and Chitosan. Mar. Drugs..

[44] Chai H., Lin Y., Evans D.G., Li D. (2008). Synthesis and UV absorption properties of 2-naphthylamine-1, 5-disulfonic acid intercalated Zn−Al layered double hydroxides. Ind. Eng. Chem. Res..

[45] Zhang Y.Q., Xue C.H., Xue Y., Gao R.C., Zhang X.L. (2005). Determination of the degree of deacetylation of chitin and chitosan by X-ray powder diffraction. Carbohydr. Res..

[46] Yen M.T., Mau J.L. (2007). Physico-chemical characterization of fungal chitosan from shiitake stipes. LWT-Food. Sci. Technol..

[47] Bangyekan C., Aht-Ong D., Srikulkit K. (2006). Preparation and properties evaluation of chitosan-coated cassava starch films. Carbohydr. Polym..

[48] Prashanth K.V.H., Tharanathan R.N. (2003). Studies on graft copolymerization of chitosan with synthetic monomers. Carbohydr. Polym..

[49] Tzompantzi F., Mantilla A., Banuelos F., Fernandez J.L., Gomez R. (2011). Improved Photocatalytic Degradation of Phenolic Compounds With ZnAl Mixed Oxides Obtained from LDH Materials. Top. Catal..

[50] Xue X.Y., Zhang S.H., Zhang H.G. (2015). Structures of LDHs Intercalated with Ammonia and the Thermal Stability for Poly (vinyl chloride). Am. J. Anal. Chem..

[51] Bonev B., Hooper J., Parisot J. (2008). Principles of assessing bacterial susceptibility to antibiotics using the agar diffusion method. J. Antimicrob. Chemother..

[52] Kulikov S.N., Chirkov S.N., Il’ina A.V., Lopatin S.A., Varlamov V.P. (2006). Effect of the molecular weight of chitosan on its antiviral activity in plants. Prik. Biokhim. Mikrobiol..

[53] Jia Z., shen D., Xu W. (2001). Synthesis and antibacterial activities of quaternary ammonium salt of chitosan. Carbohydr. Res..

[54] Li M., Sultanbawa Y., Xu Z.P., Gu W., Chen W., Liu J., Qian G. (2018). High and long-term antibacterial activity against *Escherichia coli* via synergy between the antibiotic penicillin G and its carrier ZnAl layered double hydroxide. Colloid. Surf. B.

[55] Velázquez-Herrera F.D., Fetter G., Rosato V., Pereyra A.M., Basaldella E.I. (2018). Effect of structure, morphology and chemical composition of Zn-Al, Mg/ZnAl and Cu/Zn-Al hydrotalcites on their antifungal activity against *A. niger*. J. Environ. Chem. Eng..

[56] Mishra G., Dash B., Pandey S., Sethi D., Kumar C.G. (2017). Comparative Evaluation of Synthetic Routes and Antibacterial/Antifungal Properties of Zn–Al Layered Double Hydroxides Containing Benzoate Anion. Environ. Eng. Sci..

[57] Li M., Zhu L., Lin D. (2011). Toxicity of ZnO nanoparticles to *Escherichia coli*: Mechanism and the influence of medium components. Environ. Sci. Technol..

[58] Colinas I.R., Rojas-Andrade M.D., Chakraborty I., Oliver S.R.J. (2018). Two structurally diverse Zn-based coordination polymers with excellent antibacterial activity. CrystEngComm.

[59] Zou Y.H., Wang J., Cui L.Y., Cui R.C., Wang Q.Z., Han Q.X., Qiu J., Chen X.B., Chen D.C., Chen S.K. (2019). Corrosion resistance and antibacterial activity of zinc-loaded montmorillonite coatings on biodegradable magnesium alloy AZ31. Acta Biomater..

[60] Bahrani S., Hashemi S.A., Mousavi S.M., Azhdari R. (2019). Zinc-based metal-organic frameworks as nontoxic and biodegradable platforms for biomedical applications: Review study. Drug Metab. Rev..

[61] Pasquet J., Chevalier Y., Pelletier J., Couval E., Bouvier D., Bolzinger M.A. (2014). The contribution of zinc ions to the antimicrobial activity of zinc oxide. Colloids Surf. A Phys. Eng. Asp..

[62] Chang A.K.T., Frias R.R., Alvarez L.V., Bigol U.G., Guzman J.P.M.D. (2019). Comparative antibacterial activity of commercial chitosan and chitosan extracted from *Auricularia* sp.. Biocatal. Agric. Biotechnol..

[63] No H.K., Park N.Y., Park S.H., Meyers S.P. (2002). Antibacterial activity of chitosans and chitosan oligomers with different molecular weights. Int. J. Food. Microbiol..

[64] Andres Y., Giraud L., Gerente C., Le Cloirec P. (2007). Antibacterial Effects of Chitosan Powder: Mechanisms of action. Environ. Technol..

[65] Tanyildizi M.S., Özer D., Elibol M. (2005). Optimization of α-amylase production by *Bacillus* sp. using responsesurface methodology. Process Biochem..

[66] Zeng H.Y., Deng X., Deng Y.J., Liao K.B. (2009). Preparation of Mg-Al Hydrotalcite by Urea Method and Its Catalytic Activity for Transesterification. AIChE J..

[67] Liao E.I., Minussi F.B., Da Cruz C.C.T., Bernardi A.C.C., Ribeiro C. (2012). Urea-Montmorillonite-Extruded Nanocomposites: A Novel Slow-Release Material. J. Agric. Food Chem..

[68] Chi Y.S., Shu-ning W.S. (2008). Influence of Different Al Resources and Preparation Methods on the Synthesis of Mg-Al Hydrotalcite-like Compounds. Plast. Addit..

[69] Park K.S., Kang K.T., Lee S.B., Kim G.Y., Park Y.J., Kim H.G. (2004). Synthesis of LiFePO_4_ with fine particle by co-precipitation method. Mater. Res. Bull..

[70] Bredin J., Regli A.D., Pages J.M. (2005). Propyl paraben induces potassium efflux in *Escherichia coli*. J. Antimicrob. Chemother..

[71] Schaechter M., Maaløe O., Kjeldgaard N.O. (1958). Dependence on medium and temperature of cell size and chemical composition during balanced growth of *Salmonella typhimurium*. J. Gen. Microbiol..

